# Correction to: Osimertinib as first-line therapy in advanced NSCLC: a profile of its use

**DOI:** 10.1007/s40267-018-0594-z

**Published:** 2018-12-27

**Authors:** Lesley J. Scott

**Affiliations:** Springer, Private Bag 65901, Mairangi Bay, 0754 Auckland, New Zealand

## Correction to: Drugs & Therapy Perspectives 2018 34(8):351–357 10.1007/s40267-018-0536-9

The article Osimertinib as first-line therapy in advanced NSCLC: a profile of its use, written by Lesley J. Scott, was originally published Online First without open access. After publication in volume 34, issue 8, pages 351–357, AstraZeneca Pharmaceuticals LP requested that the article be Open Choice to make the article an open access publication. Post-publication open access was funded by AstraZeneca Pharmaceuticals LP. The article is forthwith distributed under the terms of the Creative Commons Attribution-NonCommercial 4.0 International License (http://creativecommons.org/licenses/by-nc/4.0/), which permits any noncommercial use, duplication, adaptation, distribution and reproduction in any medium or format, as long as you give appropriate credit to the original author(s) and the source, provide a link to the Creative Commons license and indicate if changes were made.

The original article has been corrected.

